# Review of Handbook of Physics in Medicine and Biology, edited by Robert Splinter

**DOI:** 10.1186/1475-925X-9-53

**Published:** 2010-09-18

**Authors:** Ivan A Dotsinsky

**Affiliations:** 1 Center of Biomedical Engineering, Bulgarian Academy of Sciences, 105, Acad. G. Bonchev Str., 1113 Sofia, Bulgaria

## Competing interests

The author declares that they have no competing interests.

